# Towards an understanding of the control of ‘crumbly’ fruit in red raspberry

**DOI:** 10.1186/s40064-015-1010-y

**Published:** 2015-05-15

**Authors:** J. Graham, K. Smith, S. McCallum, P. E. Hedley, D. W. Cullen, A. Dolan, L. Milne, J. W. McNicol, C. A. Hackett

**Affiliations:** James Hutton Institute, Dundee, Scotland DD2 5DA UK; Biomathematics and Statistics Scotland, Dundee, Scotland DD2 5DA UK

**Keywords:** Raspberry, Crumbly fruit, QTL mapping

## Abstract

The genetic disorder known as ‘crumbly’ fruit is becoming a serious problem in the European raspberry industry. The study set out to examine the crumbly phenotype in a red raspberry mapping population under two environments (field and polytunnel) across six seasons in an effort to understand variability of the syndrome and to examine whether genetic factors were important and if so, whether QTL associated with the phenotype could be identified. This highlighted that seasonal, environmental (field or polytunnel) and genetic factors all influence the condition. Two QTL that are important for the genetic control of the condition have been located on linkage groups one and three, and an association with ripening time has been identified.

**Electronic supplementary material:**

The online version of this article (doi:10.1186/s40064-015-1010-y) contains supplementary material, which is available to authorized users.

## Background

Raspberry fruits are formed from an aggregation of multiple fertilized ovaries each of which are referred to as drupelets as they become fleshy. In the condition known as crumbly fruit, which has been linked with pollen abortion and embryo sac degeneration, drupelets are generally reduced in number but greatly enlarged or, in the case of small reductions, cohere imperfectly so fruit readily crumbles when picked (Daubeny et al. 1967; Jennings 1988).

Crumbly fruit is an indication of a partial failure in one or more physiological processes concerned with fruit development (Jennings 1967b) and is an increasing problem for the European raspberry industry, with particular problems occurring in widely grown commercial cultivars Tulameen and Glen Ample. There have been a number of causes suggested for the crumbly condition. It is known that infection with certain viruses can increase the likelihood that plants become crumbly (Jennings 1988). Raspberry Bushy Dwarf Virus (RBDV) infects pollen, reducing its capacity to induce fruit-set and can lead to failure of almost half of all drupelets to set (Murant et al. 1974; Daubeny et al. 1978). A genetic cause has been demonstrated where the crumbly phenotype arises from virus-tested mother plants (Jennings 1988). Studies have also shown that extensive tissue culturing of plants may increase the emergence of the condition (N. Jennings pers comm.). Additionally, environmental factors such as low or high temperatures at particular time points in development appear to play an important role with variations in the extent of crumbliness apparent from year to year (A. Dolan pers comm).

Assessment of the fruit of mother plants is currently the only method of detecting crumbliness in nuclear stock material, and it has been observed that a small number of plants with varying degrees of crumbliness can be detected each year. Some cultivars appear to be more prone to the condition than others. However if the environmental conditions differ from the normal seasonal levels, it has been observed that random symptoms of crumbliness can be displayed in cultivars not previously known for the problem. Also, known crumbly affected cultivars can show more extreme symptoms.

This material is not released to industry but may result, unneccessarily, in a cultivar permanently losing its position in the market place.

The genetic basis of raspberry fruit development is not well understood, although some studies have been carried out to look at overall control of fruit development and ripening (Graham et al. 2009) and also specific ripening related processes such as anthocyanin production (Kassim et al. 2009) colour development (McCallum et al. 2010) and volatile production (Paterson et al. 2013).

The current model of regular fruit set implies that ovary growth is blocked before pollination and that auxin is a key regulator of ovary growth de-repression at fruit set (Goetz et al. 2007; Pandolfini et al. 2007). Auxin responsive protein IAA9 and auxin response factor ARF8 repress ovary growth before fertilisation. Following pollination in raspberry there is a period of rapid growth due to cell division. This is followed by a period of slow growth during which the embryo develops and the endocarp becomes hardened, until finally cell enlargement results in a period of rapid growth. Other phytohormones (giberellin, cytokinin, brassinosteroids, ethylene and abscisic acid) play a role in fruit initiation and development (Schwabe and Mills 1981; Vriezen et al. 2008). Delayed differentiation of the embryo sac has been associated with low drupelet set in some clones of the diploid cultivar Sumner. The cultivar Latham can also show a crumbly phenotype and this is thought to be due to mutation of the dominant allele at a heterozygous gene locus causing plants to become homozygous for a deleterious recessive gene (Jennings 1967b). In Sumner it has been suggested that the effects on the embryo sac and also the reduced production of fertile pollen are caused by a mutation giving homozygosity for two recessive gene pairs (Daubeny et al. 1967).

Jennings (1967a) suggested that in cultivar Norfolk Giant embryo sac development ceased at an early stage. Jennings (1971) also suggested that an optimum status for a maternal growth substance was required for good fruit set and seed development. However there was evidence that the strength of maternal effects was considerably influenced by environmental factors.

From a cv. Latham self, Jennings (1967b) demonstrated that seedlings obtained could be classified into three groups: normal, crumbly and sterile. He proposed a model of two genetic loci, designated *St* for one whose recessive form gives complete sterility and *Cr* whose recessive form gives crumbly fruit, to explain the 9:3:4 segregation ratio obtained of normal (StCr) : crumbly (Stcr) : sterile (stCr or stcr) as *st* is epistatic to *Cr. Cr* were postulated to be linked to *Gene H* (pubescent canes) and also *gene T* (fruit colour) (Jennings, 1988). *Gene H* has been identified in raspberry on linkage group 2 (Graham et al. 2006) and a QTL for fruit colour was also identified close to *Gene H* (McCallum et al. 2010). This may suggest *Cr* is as Jennings suggested also on this chromosome.

This work set out to examine whether the crumbly fruit syndrome segregated in a population using Latham as one parent and to examine the seasonal and environmental impact on expression of the crumbly trait and identify QTL associated with the crumbly phenotype. It also set out to identify any association with the *Gene H* region and impact of genes for fertility on the trait.

## Results and discussion

### Phenotypic scoring

Over a period of 7 fruiting seasons we have investigated the segregation of crumbly fruit syndrome in a Latham x Glen Moy cross. The results show a complex pattern of two ‘crumbly’ phenotypes basically differing in severity, one of which may be the sterile phenotype proposed by Jennings (1967b). Table 1 shows the proportion of the offspring with crumbly fruit for each year and environment. The highest proportion occurred in the field in 2011, where 73 % of the offspring had crumbly fruit. The lowest proportion of crumbly fruit in the field was in 2008, where only 4 % were scored as crumbly. The proportions of crumbly fruit were always lower in the polytunnel than in the field, ranging from 22 % in 2010 to 1 % in 2008. In 2010 the crumbly and sterile phenotypes were scored separately, for a single replicate of the 188 lines of the mapping population. In the field, 115 were scored as normal, 50 as crumbly and 15 as sterile, with 8 missing scores. In the polytunnel, 117 were scored as normal, 29 as crumbly and 5 as sterile, with 37 missing scores. Comparing the two environments, 87 were scored as normal in both field and polytunnel in 2010, 15 were scored as crumbly in both environments and 3 were scored as sterile in both environments. In view of the small number of plants classed as ‘sterile plants’ and the lack of agreement in scoring this between environments we have combined the sterile and crumbly classes in further analyses.

**Table 1:** Proportion of the offspring with crumbly fruit

Year	Env	No. scored	Proportion crumbly fruit	s.e.
2004	Field A	94	0.54	0.051
2004	Field B	94	0.31	0.048
2007	Field A	188	0.27	0.032
2007	Poly	188	0.09	0.021
2008	Field A	188	0.04	0.015
2008	Poly	188	0.01	0.007
2009	Field A	188	0.32	0.036
2009	Poly	188	0.04	0.007
2010^a^	Field A	180	0.36	0.036
2010^a^	Poly	153	0.22	0.034
2011	Field A	168	0.73	0.035
2012	Field A	142	0.64	0.040
Severity (0–4)			mean	s.e.
2011	Field A	168	1.16	0.077
2012	Field A	142	1.16	0.093

2009 values are based on 3 replicates, others on one. s.e. = standard error

^a^2010 shows the proportion of either crumbly or sterile fruit

Table 2 shows gamma statistics measuring associations between the field crumbly scores for the different years. The polytunnel scores were excluded here due to the low incidence of crumbliness. The gamma statistics showed that there were no significant associations (*p* > 0.05) of other years with the 2008 scores, which had the lowest level of crumbliness. The associations of other years with the 2004 scores, which were based on MP1 only, were also generally small. The associations among the scores on MP1 and MP2 in 2007, 2009, 2010, 2011 and 2012 were all highly significant (gamma ≥ 0.67, *p* < 0.001), with a particularly high gamma statistic of 0.99 between the severity scores in 2011 and 2012.

**Table 2:** Gamma statistics for associations among the crumbly scores from the field sites

	2004, Field A	2004, Field B	2007	2008	2009	2010	2011
2004, Field B	0.59*						
2007	0.40	0.71**					
2008	0.26	0.65	−0.04				
2009	0.62**	0.54*	0.97***	0.34			
2010	0.62*	0.81***	0.81***	0.42	0.79***		
2011	0.48*	0.57*	0.78***	0.05	0.70***	0.78***	
2012	0.72***	0.61*	0.75***	0.08	0.67***	0.80***	0.99***

2009 values are based on 3 replicates, others on one. The severity scores are used in 2011 and 2012

*** *p* < 0.001; ** *p* < 0.01; * *p* < 0.05

No progeny were always scored as crumbly, though some were assessed as being crumbly 75 % or more of the times scored. Some individuals never exhibited the crumbly phenotype. The crumbly phenotype was always more severe under the open field conditions than in the polytunnel and varied significantly from season to season, according to the over years and sites analysis. As well as the environmental and seasonal effect, the agreement over years measured by the gamma scores indicated a strong genetic effect for the crumbly trait. Met Office monthly weather data was examined on maximum and minimum temperature, frost, rain and sunshine but no associations could be identified between weather conditions and extent of the crumbly condition across seasons. For example in the two severe seasons 2004 and 2012 the weather conditions were very different, with 2004 being a warm dry season and 2012 cooler and wetter. There was a late spring frost in 2012 which did not occur in 2004. In 2008 where little crumbly fruit occurred, the major difference was in the amount of rainfall at the open flower stage.

### Linkage mapping and QTL analysis

#### Mapping and QTL analysis

**Fig. 1:**
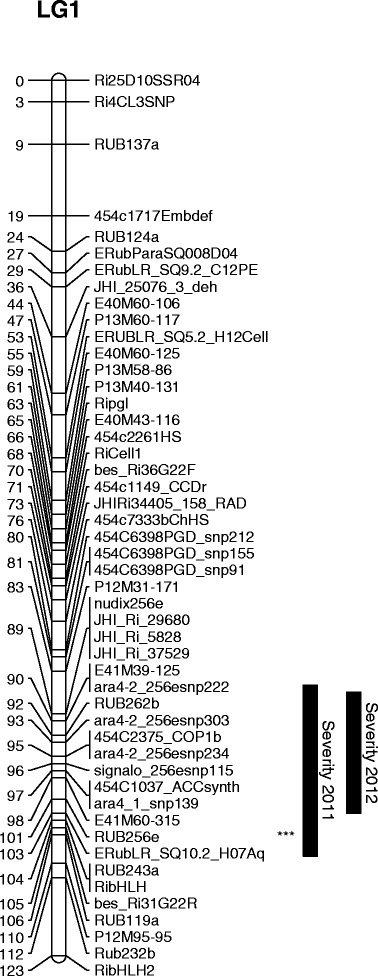
Linkage map for LG 1. The most significant marker according to the Kruskal-Wallis test is the same for all binary traits, and is shown by ***. One-lod support intervals for the severity traits are also shown

Based on permutation tests, a threshold of 13.8 for the Kruskal-Wallis (KW) statistic with one degree of freedom was used, corresponding to a genome-wide significance of *p* = 0.05. The Kruskal-Wallis analysis indicated associations above this threshold of the crumbly phenotypes with markers on LG 1 for the field scores in 5 of the 7 seasons analysed (2007, 2009, 2010, 2011 and 2012). No significant associations with this region were detected in the field trials in 2004 (the first full fruiting year) or in 2008, when the incidence of crumbly was very low (4 %), or with any of the polytunnel trials, which had a much lower incidence of the condition. The most significant region included marker RUB256e, an SSR with four alleles (ab in Latham, cd in Glen Moy) at 101 cM, although typically markers between 90 cM and 110 cM were significant. Figure 1 shows the linkage map of LG 1, with the most significant marker indicated along with one-LOD support intervals for the severity scores. Table 3 shows the results of modelling the relationship between each of the crumbly traits and this marker, using a generalised linear model with binomial errors and a logit link function for the binary scores and a normal model for the severity scores, and expressing the marker effects as additive effects of each parent together with a dominance effect, as defined in equation (1). For the field scores from 2007, 2009, 2010, 2011 and 2012 (ie where the Kruskal-Wallis test was above the genome-wide permutation threshold), the additive effect of the Latham parent was significant (*p* < 0.001) in the GLM, but the additive effect of the Moy parent and the dominance effect were not significant (*p* > 0.05). For each of these traits the direction of the effect was consistent, with the mean proportion of crumbly fruit being significantly higher in the genotypes carrying the Latham ‘b’ allele than in those with the Latham ‘a’ allele. For the field scores from 2004 and the polytunnel scores from 2007 and 2009, the additive effect of the Latham allele at RUB256e was significant (0.005 < *p* < 0.05) but again the additive effect of the Moy parent and the dominance effect were not significant (*p* > 0.05). Again, the mean proportion of crumbly fruit was higher in the genotypes carrying the Latham ‘b’ allele than in those with the Latham ‘a’ allele. The last two columns of Table 3 show the predicted proportion of crumbly fruit in the two genotype classes.

**Table 3:** Effect of the RUB256e marker on LG 1 on the crumbly scores

Year	Env.	KW statistic (3 df)	Sig. of Latham allele	Mean crumbly score for a- offspring (s.e.)	Mean crumbly score for b- offspring (s.e.)
Incidence (0–1)					
2004	Field A	4.7	0.046	0.44 (0.071)	0.65 (0.071)
2004	Field B	7.4	0.007	0.18 (0.056)	0.44 (0.074)
2007	Field A	26.9***	< 0.001	0.12 (0.033)	0.43 (0.053)
2007	Poly	5.8	0.036	0.05 (0.022)	0.14 (0.037)
2008	Field A	0.5	0.849	0.04 (0.019)	0.05 (0.022)
2008	Poly	5.7	0.078	0.00 (0.001)	0.02 (0.016)
2009	Field A	20.8***	< 0.001	0.16 (0.037)	0.49 (0.054)
2009	Poly	5.6	0.005	0.02 (0.007)	0.06 (0.012)
2010	Field A	25.1***	< 0.001	0.22 (0.042)	0.53 (0.055)
2010	Poly	4.4	0.094	0.17 (0.041)	0.28 (0.053)
2011	Field A	19.7***	< 0.001	0.58 (0.052)	0.89 (0.036)
2012	Field A	24.4***	< 0.001	0.46 (0.059)	0.83 (0.044)
Severity (0–4)					
2011	Field A	27.8***	< 0.001	0.77 (0.097)	1.59 (0.104)
2012	Field A	26.3***	< 0.001	0.69 (0.121)	1.62 (0.121)

KW = the Kruskal-Wallis statistic for this marker; df = degrees of freedom. The last three columns show the significance of the additive effect of the Latham allele in a generalised linear model, and the predicted mean crumbly score for the offspring inheriting either the ‘a’ allele or the ‘b’ allele from Latham

*** *p* < 0.001

**Fig. 2:**
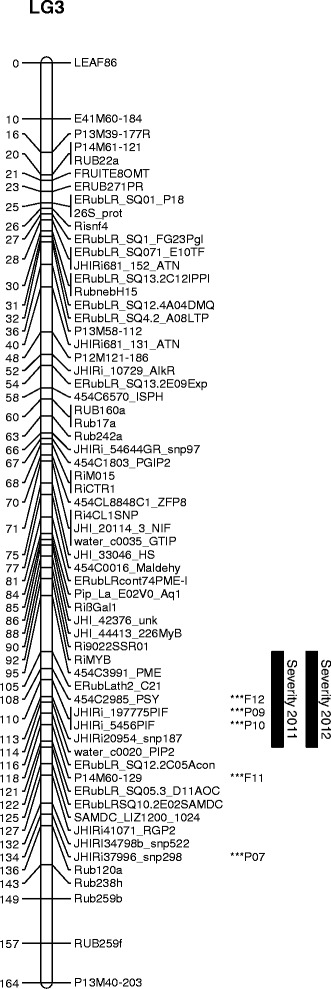
Linkage map for LG 3. The most significant markers according to the Kruskal-Wallis test are shown by *** for each binary trait, together with year and site. One-lod support intervals for the severity traits are also shown

The Kruskal-Wallis analysis also indicated associations above the genome-wide permutation threshold between the crumbly scores from the polytunnel in 2007, 2009 and 2010 and the field severity scores from 2011 and 2012 with markers segregating on LG 3, although the best marker varied slightly (region 107-133 cM). Again the significant markers segregated in the Latham parent. Figure 2 shows the linkage map of LG 3, with the most significant marker for each trait indicated along with one-LOD support intervals for the severity scores. The marker ERubLR_SQ05.3_D11AOC at 121 cM was chosen as representative of this region, on the grounds of a low number of missing scores, and was included together with RUB256e from LG 1 in a further GLM to test their joint significance. The significance of the additive effect of the Latham parent at marker ERubLR_SQ05.3_D11AOC for the traits above was confirmed, with *p* = 0.003 for the polytunnel in 2007 and *p* < 0.001 for the other traits. ERubLR_SQ05.3_D11AOC also showed a lower significance in the GLM for traits 2004 Field B (*p* = 0.003), 2010 Field (*p* = 0.004), 2011 Field incidence (*p* = 0.017) and 2012 Field incidence (Field = 0.011). For all of these traits the direction of the effect was consistent, and there was no significant interaction (*p* > 0.05) between the two markers ERubLR_SQ05.3_D11AOC and RUB256e. Table 4 shows the predicted mean severity scores in 2011 and 2012 from the GLM for the two markers together, ranging from 0.45 to 2.0.

**Table 4:** Predicted means (se) at both loci for the severity scores using a two-marker model

Year	LG1 = a-; LG3 = a-	LG1 = a-; LG3 = b-	LG1 = b-; LG3 = a-	LG1 = b-; LG3 = b-
2011	1.23 (0.128)	0.53 (0.102)	2.00 (0.125)	1.29 (0.113)
2012	1.16 (0.158)	0.45 (0.128)	2.00 (0.145)	1.29 (0.138)

LG1 is represented by RUB256e and LG3 by ERubLR_SQ05.3_D11AOC

A combined analysis over years and sites (field or polytunnel) was conducted on the binary scores, omitting the data from 2004 as this was only scored on the MP1 lines. The analysis of deviance table for the incidence of crumbliness is shown in Additional file 1: Table S1. This shows significant effects of year, site and their interaction and significant effects of the Latham parent at the markers RUB256e on LG 1 and ERubLR_SQ05.3_D11AOC on LG 3. There were no significant interactions involving year, but there was a significant interaction between site and each of the markers. Table 5 shows the mean crumbly scores from these interactions, with the effect of RUB256e on LG 1 being greater at the field sites and that of ERubLR_SQ05.3_D11AOC on LG 3 being greater in the polytunnel sites. A similar combined analysis was conducted on the 2011 and 2012 field severity scores together, but no significant interactions between the year and the marker were detected.

**Table 5:** Predicted means (se) for the crumbly scores from the generalised linear model combining data over years and sites

(a) Site.LG1 interaction		
Site	LG1 = a-	LG1 = b-
Field	0.17 (0.013)	0.51 (0.020)
Poly	0.05 (0.009)	0.10 (0.013)
(b) Site.LG3 interaction		
Site	LG3 = a-	LG3 = b-
Field	0.35 (0.018)	0.30 (0.015)
Poly	0.13 (0.016)	0.04 (0.008)

LG1 is represented by RUB256e and LG3 by ERubLR_SQ05.3_D11AOC

### Relationship with ripening

The crumbly scores in 2007, 2009, 2010, 2011 and 2012 showed significant correlations (*p* < 0.05) with some of the time to ripening scores recorded for this population in 2006 by Graham et al. (2009), as shown in Table 6. The largest correlations were with the time to reach the fruit set stage and the time to reach green fruit. Some crumbly scores also had a significant correlation with the time to reach the green/red stage, but the time to reach the open flower stage was not correlated with the crumbly scores. The correlations were positive i.e. the proportion of crumbly fruit increases with the time taken to reach fruit set and green fruit. Ripening is also associated with many markers on LG 3 including the region identified above (Graham et al. 2009). A GLM with markers ERubLR_SQ05.3_D11AOC and RUB256e and time to fruit set was investigated using all-subset regression to identify the most significant explanatory variables for each crumbly score, but there was no consistency in the choice among time to fruit set, ERubLR_SQ05.3_D11AOC or both of these. We cannot therefore draw any conclusions at present as to whether ripening time affects crumbliness directly or whether both traits are controlled by one or more genes on LG 3. The association with ripening is interesting, with the longer the fruit takes to get to the fruit set and green fruit stage, the more likely it is to be crumbly. This is particularly apparent when considering the difference between polytunnel grown fruit and field grown fruit where under field conditions the fruit always take longer to get to these stages and beyond. Graham et al. (2009) identified markers on LG 3 as associated with time to ripening. At this stage however conclusions cannot be drawn as to whether ripening time affects crumbliness directly or whether both traits are controlled by one or more genes on LG 3. Contrary to the suggestion by Jennings (1967b) that crumbly fruit was related to the Gene H region, no genetic association with this region on LG 2 could be identified with the crumbly fruit syndrome. The Gene H region is an interesting region associated with a number of diverse traits (Knight and Keep 1958; Jennings and Brydon 1989; Jennings 1962; Keep 1968, 1976; Jennings and McGregor 1988; Anthony et al. 1986; Jennings 1967a). Interestingly, the *Hh* genotype of Gene H was associated with a slowing down of ripening across all stages from open flowers to the green/red stage compared to the *hh* genotype (Graham et al. 2009). The correlation with Gene H and crumbly fruit identified by Jennings may actually be due to this association with ripening time rather than to the region itself. Interestingly alleles associated with longer time to ripening in the Gene H region and also on LG 3 and LG 5 are associated with smaller root density and diameter measures and may be regarded as general vigor genes (Graham et al. 2011). This may also be a factor in crumbly fruit and will need further investigation.

**Table 6:** Correlation between crumbly scores and the time to each of the ripening stages from 2006

Year	Env	Open	Fruit set	Green	Green/Red	Ripe
2004	Field A	−0.18	0.19	0.17	0.14	0.08
2004	Field B	−0.03	0.11	0.11	−0.09	0.11
2007	Field A	−0.05	0.26***	0.29***	0.14	0.08
2007	Poly	−0.15	0.16*	0.10	0.21**	0.07
2008	Field A	−0.09	−0.02	−0.11	−0.12	0.00
2008	Poly	−0.06	0.04	−0.01	0.04	0.04
2009	Field A	−0.05	0.30***	0.32***	0.17*	0.07
2009	Poly	0.01	0.22**	0.14	0.00	−0.17*
2010^a^	Field A	−0.01	0.31***	0.27***	0.17*	0.10
2010^a^	Poly	−0.05	0.30***	0.27***	0.18*	−0.06
2011	Field A	−0.07	0.18*	0.17*	0.14	0.02
2012	Field A	−0.13	0.21*	0.22**	0.23**	0.08
Severity (0–4)						
2011	Field A	−0.03	0.29***	0.29***	0.20*	0.01
2012	Field A	−0.02	0.29***	0.29***	0.24**	0.01

^a^2010 shows the proportion of either crumbly or sterile fruit

*** *p* < 0.001** p < 0.01; * p < 0.05

### Gene content in Rub256e region

The raspberry genome pseudomolecules (provided by Joshua Udall BYU, Genetics and Biotech Faculty (pws.byu.edu)) were searched using BLAST (Altschul et al. 1990) for any regions that matched RUB256e. Six different genes were predicted in the region as follows: Methyl transferase (XP004133879.1), signalosome complex (XP002511799.1), cysteine protease (XP002306369.1), Ara4 interacting protein (XP002511798.1), Nudix hydrolase.

(XP002266987.1) and Methyl transferase (AER13155.1) containing the Rub256e marker and those containing a polymorphism between the parents were mapped using primers in Table 7 to confirm location and allow future gene expression studies to be carried out.

**Table 7:** Primers to confirm location of genes in Rub 256e region

Gene in Rub256e region	Primer sequence
Ara4 1 256e	Ggcaagtttacccagctgaa
	catatgagtgcgcagatacag
Ara4 2 256e	Cattccctgcgttgaaatct
	Ttctgagtcgtctggtgtgc
Nudix256e	Gaaggttttcggtaccacca
	tcctgcttctggatgtcaaa
Signalo256e	Tgcatcctggatatggattt
	ccaagttgcccatgagaataa

## Conclusion

This study has highlighted that environmental, seasonal and genetic factors all play a role in the development of crumbly fruit in red raspberry. A region on LG 1 at the Rub256e marker, and an association with ripening time and ripening associated markers on LG 3 were identified for further analysis. No association with crumbly fruit and Gene H was determined. This work has allowed us to identify a genetic component to the condition which can be assessed for breeding lines less prone to crumbly fruit. Controlled environment studies will be carried out in an attempt to define triggers of the condition in those samples where the phenotype varies between seasons and environments.

## Materials and methods

### Field and polytunnel trials

The population, as described previously (Graham et al. 2004, 2006, 2009, 2011, 2014; Woodhead et al. 2013), consists of a full sib family generated from a cross between the European red raspberry cv. Glen Moy and the North American red raspberry cv. Latham. Trials were arranged in a randomised block design with three replicates each containing two replicated plants of 330 genotypes at two field sites, and three single-plant replicates of 188 genotypes (randomly selected from the original 330 full sib family for mapping purposes) under polythene tunnel protection (McCallum et al. 2010).

### Phenotypic data collection for crumbly fruit

Mapping of this population has focused on two subsets, an initial population mapping population 1 (MP1) of 94 seedlings, and a further mapping population of an additional 94 seedlings (MP2). Phenotypic data on crumbly fruit, scored as crumbly or not according to Jennings 1967b, was collected on MP1 only in 2004 on a single replicate at two field locations (A & B). In 2007 and 2008, crumbly fruit was assessed similarly on a single replicate of the lines in MP1 and MP2 at one field site (A) and on plants grown under a polytunnel. In 2009, three replicates were assessed for the lines in MP1 and MP2 at both field sites (A & B) and in the polytunnel. In 2010, a single replicate of MP1 and MP2 was assessed at one field site (A) and in the polytunnel, but the scoring was modified to distinguish between crumbly and a more severe form where no drupe development occurred, which we referred to as ‘sterile fruit’. This was examined to see if it showed any association with sterility as described previously (Jennings 1967b) or was an extension of the crumbly fruit phenotype. In 2011 and 2012 a single replicate of MP1 and MP2 was scored at the field site (A) only; for these years crumbliness was scored as both crumbly or not and on a 0–4 scale where 0 was no crumbly fruit and 4 was the severe ‘sterile’ condition.

Raspberry Bushy Dwarf Virus (RBDV) testing was carried out as standard to ensure plants were free of the virus (http://www.fruithealth.co.uk).

### Linkage mapping, summary statistics and QTL analysis

Previous versions of the linkage map for this population have been described by Graham et al. (2004, 2006, 2009, 2011, 2014), McCallum et al. (2010) and Woodhead et al. (2010, 2013). Further markers have been added to the map used here, using JoinMap 4.1 (Van Ooijen 2006) Table 7.

As the crumbly scores are binary or ordinal traits, associations between them were calculated using the gamma statistic (Siegel and Castellan 1988), which varies between −1 and +1.

The QTL mapping analyses were chosen to be suitable for binary and ordinal traits. A non-parametric mapping based on the Kruskal-Wallis (KW) test was used initially to test each marker on the map for associations with the crumbly fruit scores for each year and environment, using the MapQTL 5 software (Van Ooijen 2004). The KW test statistic has an approximate chi-square distribution with degrees of freedom equal to the number of genotype classes minus one under the hypothesis of no segregating QTL. The threshold for the Kruskal-Wallis mapping across the genome was established using a small permutation test of 400 permutations (carried out in GenStat 16 for Windows (Payne et al. 2013)). For a normally distributed trait, the subsequent analysis would be to combine information across genetic markers along the chromosome to estimate the probabilities of each possible QTL genotype for each offspring at each position (the “genetic predictors”) and to model each trait as a function of these using a standard linear model. In a cross such as this with outbreeding parents, the parental genotypes at a QTL are usually represented as ab x cd, with offspring genotypes ac, ad, bc and bd and the probabilities for these genotypes can be used in the linear model. Alternatively genetic predictors for the maternal additive effect (*P*_*1*_*)*, the paternal additive effect (*P*_*2*_) and the dominance effect (*D*) can be derived for each offspring at each position as:

1 $$ \begin{array}{l}{P}_1=pr(bc)+pr(bd)-pr(ac)-pr(ad)\\ {}{P}_2=pr(bd)+pr(ad)-pr(bc)-pr(ac)\\ {}D=pr(bd)-pr(bc)-pr(ad)+pr(ac)\end{array} $$

where *pr(ac)* is the probability that the offspring has genotype ac at that position, and these can be used in the linear model. For the binary traits here, the linear model was replaced by a generalised linear model (GLM) with binomial errors and a logit link function to relate the crumbly trait to the additive and dominance effects in the genetic regions identified by the KW analysis. The genetic predictors of the additive effects of each parent and the dominance effect were calculated at each marker position using the QIBDPROBABILITIES procedure of GenStat 16 for Windows (Payne et al. 2013) and this program was also used to fit the GLM. Finally a GLM analysis was carried out over the years and sites together, fitting year, site, the genetic effects and all interactions. Linear models with normally distributed errors was used for the field severity scores from 2011 and 2012.

### Identification of gene content in Rub256e region and mapping to confirm location

Little information in terms of functional markers was available for the Rub256e linkage map region on linkage group (LG) 1, therefore the raspberry genome pseudomolecules (provided by Joshua Udall BYU, Genetics and Biotech Faculty (pws.byu.edu)) were searched using BLAST (Altschul et al. 1990) for any regions that matched the RUB256e sequence. Primers were designed to some genes in the region (Table 6) and added to the linkage map as previously described (Graham et al. 2011) to confirm location of sequence.

### Association with ripening

Graham et al. (2009) studied the development of fruit in the same population and identified QTL associated with the ripening process. Here the association between ripening rates and crumbliness was investigated using the ripening field scores from 2006, as these were available for both MP1 and MP2. The ripening data was originally scored as developmental stages using a 1–7 scale (1 = bud break, 2 = open flowers, 3 = fruit set, 4 = green fruit, 5 = green/red fruit, 6 = ripe and 7 = over-ripe), with the first scoring on 19th May 2006 when all scores were equal to one. From these scores Graham et al. (2009) estimated the number of days to reach each of the developmental stages for each genotype and mapped QTL for these. The association with crumbliness was investigated here using correlation coefficients. The crumbly traits were also modelled as a function of both the genetic markers and the time to ripening, using a generalised linear model with binomial errors and a logit link function.

## Additional file

Additional file 1: Table S1. Analysis of deviance table for the incidence of crumbly fruit, modelled as a function of site, year, genetic effects and their interactions. LG1 is represented by Rub256e and LG3 by ERubLR_SQ05.3_D11AOC, using the additive effect of the Latham allele in each case.

## Abbreviations

QTL: Quantitative trait loci
MP: Mapping population
LG: Linkage group
GLM: Generalised linear model
