# Supplementary material for: Towards an understanding of the control of ‘crumbly’ fruit in red raspberry
Source: Springerplus. 2015 May 15;4:223. doi: 10.1186/s40064-015-1010-y (PMC4439414; doi:10.1186/s40064-015-1010-y)
Supplement: Additional file 1: Table S1. — Analysis of deviance table for the incidence of crumbly fruit, modelled as a function of site, year, genetic effects and their interactions. LG1 is represented by Rub256e and LG3 by ERubLR_SQ05.3_D11AOC, using the additive effect of the Latham allele in each case. [file 40064_2015_1010_MOESM1_ESM.docx]

Table S1. Analysis of deviance table for the incidence of crumbly fruit, modelled as a function of site, year, genetic effects and their interactions. LG1 is represented by Rub256e and LG3 by ERubLR_SQ05.3_D11AOC, using the additive effect of the Latham allele in each case.

| Term | d.f. | deviance | deviance  ratio | approx  chi pr |
| --- | --- | --- | --- | --- |
| Year | 5 | 367.37 | 73.47*** | <.001 |
| Site | 1 | 135.27 | 135.27*** | <.001 |
| LG1_Latham | 1 | 197.99 | 197.99*** | <.001 |
| LG3_Latham | 1 | 23.76 | 23.76*** | <.001 |
| Year.Site | 3 | 29.40 | 9.80*** | <.001 |
| Year. LG1_Latham | 5 | 6.99 | 1.40^ns^ | 0.221 |
| Site. LG1_Latham | 1 | 7.90 | 7.90** | 0.005 |
| Year. LG3_Latham | 5 | 4.04 | 0.8 ^ns^ | 0.543 |
| Site. LG3_Latham | 1 | 11.29 | 11.29*** | <.001 |
| Year.Site.LG1_Latham | 3 | 4.98 | 1.66^ns^ | 0.173 |
| Year.Site.LG3_Latham | 3 | 2.18 | 0.73^ns^ | 0.536 |
| Residual | 2091 | 1520.74 |  |  |

*** p < 0.001; ** p < 0.01;ns p > 0.05
